# Experimental and quantum chemical studies of a novel synthetic prenylated chalcone

**DOI:** 10.1186/1752-153X-7-17

**Published:** 2013-01-26

**Authors:** José C Espinoza-Hicks, Alejandro A Camacho-Dávila, Norma R Flores-Holguín, Guadalupe V Nevárez-Moorillón, Daniel Glossman-Mitnik, Luz M Rodríguez-Valdez

**Affiliations:** 1 Facultad de Ciencias Químicas. Universidad Autónoma de Chihuahua. Circuito No. 1, Nuevo Campus Universitario, Chih, Mexico; 2 Centro de Investigación en Materiales Avanzados, S.C. Blvd. Miguel de Cervantes 120, Complejo Industrial Chihuahua, Chih, Mexico

**Keywords:** NMR, Molecular structure, Quantum-chemistry methods, Flavonoid

## Abstract

**Background:**

Chalcones are ubiquitous natural compounds with a wide variety of reported biological activities, including antitumoral, antiviral and antimicrobial effects. Furthermore, chalcones are being studied for its potential use in organic electroluminescent devices; therefore the description of their spectroscopic properties is important to elucidate the structure of these molecules. One of the main techniques available for structure elucidation is the use of Nuclear Magnetic Resonance Spectroscopy (NMR). Accordingly, the prediction of the NMR spectra in this kind of molecules is necessary to gather information about the influence of substituents on their spectra.

**Results:**

A novel substituted chalcone has been synthetized. In order to identify the functional groups present in the new synthesized compound and confirm its chemical structure, experimental and theoretical ^1^H-NMR and ^13^C-NMR spectra were analyzed. The theoretical molecular structure and NMR spectra were calculated at both the Hartree-Fock and Density Functional (meta: TPSS; hybrid: B3LYP and PBE1PBE; hybrid meta GGA: M05-2X and M06-2X) levels of theory in combination with a 6-311++G(d,p) basis set. The structural parameters showed that the best method for geometry optimization was DFT:M06-2X/6-311++G(d,p), whereas the calculated bond angles and bond distances match experimental values of similar chalcone derivatives. The NMR calculations were carried out using the Gauge-Independent Atomic Orbital (GIAO) formalism in a DFT:M06-2X/6-311++G(d,p) optimized geometry.

**Conclusion:**

Considering all HF and DFT methods with GIAO calculations, TPSS and PBE1PBE were the most accurate methods used for calculation of ^1^H-NMR and ^13^C-NMR chemical shifts, which was almost similar to the B3LYP functional, followed in order by HF, M05-2X and M06-2X methods. All calculations were done using the Gaussian 09 software package. Theoretical calculations can be used to predict and confirm the structure of substituted chalcones with good correlation with the experimental data.

## Introduction

Chalcones are ubiquitous substances found in a diversity of plants. They are precursors to other natural products, such as flavonoids, which have reported a wide range of biological activities such as antioxidant, antibacterial, antitumoral among others [1]. Due to the importance of these compounds, diverse studies on synthesis and biological activities of molecules containing the chalcone ring system, have been reported recently [2]. The prenyloxychalcones are important derivatives of chalcones, which contain a prenyl-type side chain with different length, including 2,2-dimethylallyl, geranyl or farnesyl chains; these compounds have shown interesting antitumoral activity [3]. In order to obtain information on the nature and expected activities of a given organic compound, it is necessary a better understanding of its spectroscopic properties, as well as the detection of functional groups. Along chemistry community, Nuclear Magnetic Resonance (NMR) is one of the most widely used spectroscopic techniques, and the most sensitive to study organic compounds molecular structure [4-6]. On the other hand, quantum chemical calculations are an important tool for the determination of the relationship between structure and spectral properties [7]. Predicting structures and compositions of complex systems through NMR analysis by theoretical methods is a reachable goal, but an excellent accuracy of few parts per million in the calculations is necessary to obtain good precision in the calculated spectra [8].

Numerous simulation techniques have been developed for the calculation of molecular NMR shielding tensors and magnetic susceptibilities: Gauge-Independent Atomic Orbital (GIAO) formalism [9-13], Continuous Set of Gauge Transformations (CSGT) method [14-16], Individual Gauge for Local Orbitals (IGLO) method [17-21], and Localized Orbital/Local Origin (LORG) theory [22] among others. The large majority of theoretical NMR properties reported in the literature use the GIAO formalism, which is known to give satisfactory results for large molecules. Although the theoretical calculations are limited by the employed methodology, GIAO formalism shows good correlation when compared with experimental NMR values.

**Scheme 1:**
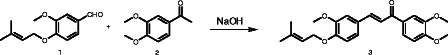
Chemical synthesis of (2E)-1-(3,4-dimethoxyphenyl)-3-{3-methoxy-4-[(3-methylbut-2-en-1-yl)oxy]phenyl}prop-2-en-1-one (Compound 3).

The aim of this work was to present the theoretical and experimental analyses of ^1^H and ^13^C NMR calculations on a novel synthesized chalcone molecule named (2E)-1-(3,4-dimethoxyphenyl)-3-{3-methoxy-4-[(3-methylbut-2-en-1-yl)oxy]phenyl}prop-2-en-1-one, compound **3**, (Scheme 1), by comparing several theoretical methods with experimental values, to attain a good accuracy in the prediction of NMR properties using GIAO formalism at both the Hartree-Fock and density functional theory (DFT) levels of theory. Also, the quantum-chemical calculations were used for a better understanding of the NMR properties as well as for an analysis of the geometrical parameters in this novel compound.

## Experimental

### Synthesis

#### General

The reaction was done under nitrogen atmosphere. Solvents employed for column chromatography were of reagent grade. Nuclear magnetic resonance spectra were recorded in diluted solutions of CDCl_3_, with a solvent containing tetramethylsilane as reference. The spectra were recorded using a Varian Spectrometer at 300 MHz. The chemical shifts are reported as parts per million (ppm).

(2E)–1-(3,4-dimethoxyphenyl)–3-{3-methoxy-4-[(3-methylbut-2-en-1-yl)oxy]phenyl}prop-2-en-1-one (3). The synthesis of compound **3** was obtained following the protocol of Liu et al. [23]. A mixture of prenyloxyvanillin **1** (1.1 g, 5 mmol) and 3, 4–Dimethoxyacetophenone **2** (1.05 g, 5 mmol) was added to a solution of 2:1 ethanol-water (40 ml) and stirred until dissolution. Then, a solution of NaOH (0.68 gr, 17 mmol) in 16 mL of water was added dropwise and the reaction mixture was stirred for 48 hours at room temperature. The reaction was extracted with ethyl acetate (50 ml), and the organic layer was washed with brine, dried (Na_2_SO_4_) and concentrated under reduced pressure. The crude product was purified by column chromatography (Silica gel Sigma-Aldrich 60 Å), eluting with hexane-ethyl acetate 9:1 to give the pure compound **3** as a yellow solid, 0.73 g. Yield: 38.17%.

^1^H-NMR, (CDCl_3 _300 MHz) δ:1.76 (s, 3H), 1.79 (s, 3H), 3.94 (s, 3H), 3.96 (s, 3H), 3.97 (s, 3H), 4.63 (s, 1H), 4.65 (s, 1H), 5.53 (m, 1H), 6.89 (d, 1H, J = 9 Hz), 6.94 (d, 1H, J = 9 Hz), 7.17 (d, 1H, J = 3Hz), 7.22 (dd, 1H, J = 9, 3 Hz), 7.42 (d, 1H, J = 15 Hz), 7.63 (d, 1H, J = 3 Hz), 7.69 (dd, 1H, J = 3, 9 Hz), 7.77 (d, 1H, J = 15 Hz). FT-IR (ATR): 2934, 1651, 1594, 1509, 1420, 1259, 1199, 1140, 1025, 982, 804, 766 cm-^1^. ^13^C-NMR (CDCl_3 _75.48 MHz) δ: 188.68, 153.11, 150.67, 149.6, 149.24, 144.27, 138.22, 131.61, 127.94, 122.87, 122.82, 119.52, 119.44, 112.65, 110.86, 110.46, 109.95, 65.81, 56.1, 56.08, 56.02, 25.87, 18.31.

### Computational details

The tested DFT methods include: two hybrid functionals (B3LYP [24,25] and PBE1PBE [26]), the meta DFT TPSS [27,28] and two hybrid meta GGA methods (M05-2X [29,30] and M06-2X [30,31]). These methods are summarized in Table 1. The DFT methods employed were also compared with the Hartree-Fock calculations. All the theoretical calculations were determined in gas phase and in the approximation of the isolated molecule, using GaussView05 program [32] and Gaussian09 [33] program package.

**Table 1:** Summary of DFT methods tested in the study

**Method**	**Type**	**Exchange/correlation functional**	**Ref**
B3LYP	Hybrid DFT	Becke88/Lee-Yang-Parr	[24,25]
PBE1PBE	Hybrid DFT	PBE/PBE	[26]
TPSS	Pure or meta DFT	TPSS/TPSS	[27,28]
M05-2X	Hybrid meta DFT	M05-2X/M05-2X	[29,30]
M06-2X	Hybrid meta DFT	M06-2X/ M06-2X	[30,31]

In order to analyze the geometrical parameters of compound **3,** the optimized geometry of the studied compound was obtained through Hartree-Fock and several DFT methods. For ground state geometry optimization of the studied chalcone **3**, these electronic structure methods were combined with a 6-311++G(d,p) basis set [34]. Frequency calculations were carried out at the same level of theory and no negative frequencies were observed. The absence of imaginary frequencies confirmed that all structures are global minima of the potential energy hypersurfaces. Calculated geometries of **3** were compared with experimental values reported for other structurally related chalcones. A statistical analysis of paired *t*-test for each method versus experimental data for specific geometrical parameters was applied. This test allowed the selection of the best method for geometry optimization. Once selected the correct theoretical geometry, NMR calculations were completed using the optimized geometry. The calculated chemical shifts of ^1^H NMR and ^13^C NMR for **3** were obtained by the GIAO method, at both, the Hartree-Fock and several DFT methods with 6-311++G(d,p) level of theory and using tetramethylsilane (TMS) as reference. It has been reported that the employment of this basis set (6-311++G(d,p)) along with several electronic structure methods, predicts chemical shifts with acceptable accuracy, and the obtained results present a good correlation between the experimental and theoretical values, especially for ^13^C [16,35-37]. Calculated ^1^H NMR and ^13^C NMR were contrasted with experimental results obtained for the new synthesized chalcone **3**. The obtained theoretical results were helpful for the detailed assignments of experimental NMR spectra of the studied compound.

## Results and discussion

### Geometry

**Figure 1:**
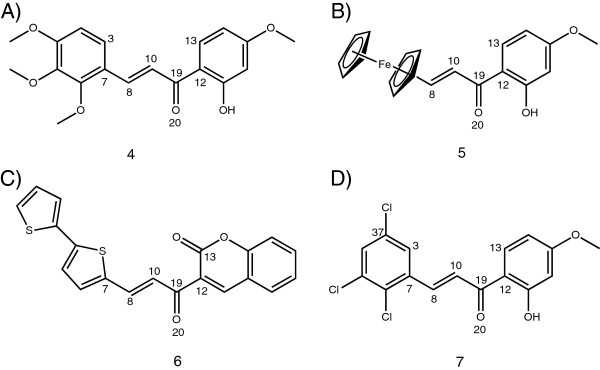
**Chemical structure and atomic numbering for chalcone derivatives with X-ray diffraction parameters reported. A)** Compound **4**, Pandi et. al [6], **B)** Compound **5**, Xiang Wu et. al. [38], **C)** Compound **6**, Sun et. al. [39], **D)** Compound **7**, Rajesh et. al. [5].

The calculated geometry for **3**, was obtained with HF, B3LYP, PBE1PBE, TPSS, M05-2X, M06-2X and 6-311++G(d,p) levels of theory. The results were compared with experimental values reported for geometrical parameters in similar chalcone derivatives. Since the X-ray crystal structure of this novel chalcone has neither been reported nor determined, we decided to use the reported X-ray analyses for similar chalcone derivatives as reference (see Table 2) [5,6,38,39]. The molecular structures of these reference compounds are shown in Figure 1.

**Table 2:** Experimental geometric parameters reported for some chalcone derivatives

**Structural parameters**	**Pandi et al. **[6]	**Xiang Wu et al. **[38]	**Sun et al. **[39]	**Rajesh et al. **[5]
**Bond length (Å)**				
C13 – C12	—	—	—	1.406
C12 – C19	—	1.496	1.501	1.472
C19 – C10	—	1.485	1.465	1.487
C10 = C8	1.323	1.324	1.321	1.319
C8 – C7	—	1.456	1.440	—
C7 – C3	—	—	—	1.377
C19 = O20	1.246	1.216	1.217	1.216
**Bond angle (º)**				
C13-C12-C19	124.0	—	123.8	118.5
C8-C7-C3	123.2	—	123.5	—
O20 = C19-C12	—	119.9	118.3	121.0
C12-C19-C10	121.8	119.2	—	120.4
O20 = C19-C10	—	120.9	—	118.6
C7-C3-C37	—	—	—	121.6
C8 = C10-C19	—	119.8	120.4	120.7
C7-C8 = C10	129.9	—	—	128.0

As observed in Table 2, the corresponding geometrical parameters for chalcones **4 **[6], **5**[38], **6**[39] and **7**[5] obtained by X-ray diffraction show similar values even though the molecules have different substituents in their structure (See Figure 1). This could indicate that the introduction of diverse functional groups into the aromatic ring system has little effect on specific geometrical parameters (prop-2-en-1-one chain), as reported in previous theoretical and experimental studies [40]. Based on the experimental data reported by Rajesh et al. [5] for chalcone **7** the reported values were used to compare with the theoretical bond lengths and bond angles in compound **3** (Table 2). Table 3 contains theoretical parameters of the optimized geometries; and as it can be observed, all tested methods showed good results for bond lengths, in particular when compared with the reported X-ray data. At this point, it was difficult to define which method is more appropriate to use for the compound under study.

**Table 3:** Geometric parameters for chalcone 3 obtained with different theoretical methods vs experimental data for chalcone 7

**Structural parameters**	**DFT:**	**DFT:**	**DFT:**	**DFT:**	**DFT:**	**HF**	**Rajesh et al. **[5]
	**B3LYP**	**PBE1PBE**	**TPSS**	**M05-2X**	**M06-2X**		
**Bond Length (Å)**							
C13 – C12	1.397	1.393	1.404	1.393	1.381	1.381	1.406
C12 – C19	1.498	1.491	1.498	1.493	1.497	1.497	1.472
C19 – C10	1.484	1.479	1.483	1.485	1.491	1.491	1.487
C10 = C8	1.346	1.343	1.355	1.342	1.328	1.330	1.319
C8 – C7	1.460	1.455	1.459	1.464	1.473	1.473	—
C7 – C3	1.406	1.402	1.413	1.403	1.396	1.396	1.377
C19 = O20	1.226	1.221	1.240	1.224	1.196	1.196	1.216
**Bond angle (º)**							
C13-C12-C19	124.18	124.23	124.27	123.51	123.83	123.84	118.5
C8-C7-C3	123.24	123.13	123.30	122.88	123.21	123.21	—
O20 = C19-C12	119.964	119.99	119.87	120.05	119.98	120.00	121.0
C12-C19-C10	118.96	118.91	119.10	118.80	118.87	118.88	120.4
O20 = C19-C10	121.07	121.10	121.05	121.15	121.14	121.14	118.6
C7-C3-C37	121.36	121.34	121.28	120.94	121.16	121.16	121.6
C8 = C10-C19	120.41	119.87	121.21	119.29	119.94	119.95	120.7
C7-C8 = C10	127.95	127.84	127.94	127.22	127.80	127.79	128.0

Additionally, a paired *t*-test analysis was applied in order to determinate which of the methods employed presents the best fit for geometry optimizations, in comparison with the corresponding experimental parameters for compound **7 **[5] (which is structurally similar to our synthesized chalcone **3)**. Statistical results, including the paired-t value and the corresponding probability (p-value) for the calculated bond lengths and bond angles with respect to experimental data are reported in Table 4. The obtained results show that only for the DFT:TPSS functional, there was a statistically significant difference between the experimental and the theoretical data (p = 0.047). On the other hand, the best results were obtained with the hybrid meta GGA M06-2X functional, which has the highest p-value for the comparison between the theoretical values and the experimental data reported for bond lengths (paired t = 0.24, p = 0.820) and bond angles (paired t = 0.33, p = 0.755). The other DFT methods predicted consistently the geometrical parameters, however, these values are not better than those obtained with M06-2X (See Table 4). It is important to note the superior performance of HF method, which shows good results in the comparison of geometric parameters with a paired t = 0.28 and p = 0.792 for bond lengths, and a paired t = 0.60 and p = 0.578 for bond angles, very similar values to the obtained results with DFT:M05-2X, particularly for bond angle data.

**Table 4:** **Statistical data of paired *t*-test for geometrical parameters calculated with several theoretical methods and compared with experimental values**

**Theoretical methods/6-311++G(d,p)**	**t-value**^**a**^	**p-value**^**b**^
**Bond lengths comparison**		
DFT:B3LYP	1.97	0.105
DFT:PBE1PBE	1.28	0.256
DFT:TPSS	2.63	0.047
DFT:M05-2X	1.65	0.161
DFT:M06-2X	0.24	0.820
HF	0.28	0.792
**Bond angle comparison**		
DFT:B3LYP	0.77	0.473
DFT:PBE1PBE	0.65	0.537
DFT:TPSS	0.89	0.406
DFT:M05-2X	0.60	0.572
DFT:M06-2X	0.33	0.755
HF	0.60	0.568

^a^ Statistic test. $t=\overset{―}{d}/\sqrt{S^{2}/n}$^b^ Estimated probability of rejecting the null hypothesis of “no difference” between experimental and theoretical values, p < 0.05.

**Figure 2:**
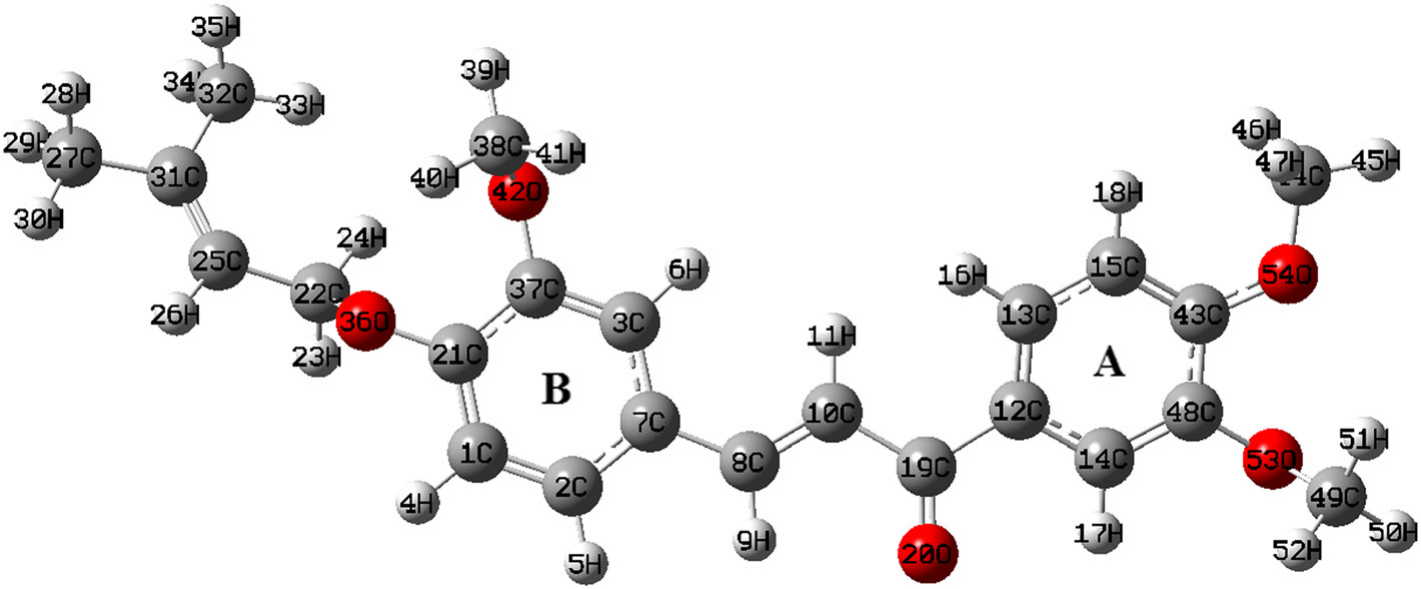
Optimized structure of chalcone 3 at DFT:M06-2X/6-311++G(d,p) level of theory.

Since the M06-2X functional has yielded excellent results for geometrical properties, this functional in combination with 6-311++G(d,p) was chosen to perform a more detailed structural analysis. Thus, according with M06-2X/6-311++G(d,p) only one potential energy hypersurface minimum was obtained (See Figure 2).

The predicted geometry shows that the prenyl side chain attached to the aromatic ring through 36O and methoxy groups, joined through 54O and 53O, are oriented in opposite directions to the B-ring–8C = 10C–19C–(20O)–A-ring, where both rings are connected through 7C and 12C atoms with the prop-2-en-1-one chain (−8C = 10C–19C–(20O)–). The computed geometry presents a nearly flat region in the central zone with a maximal deviation from total planarity of 16°, approximately. The methoxy groups at 54O and 53O are located in neighboring carbon atoms. Regarding the plane of the benzene A-ring, due to the steric hindrance, the methoxy groups are oriented at opposite sides, maintaining dihedral angles of 176.46º (48C – 43C – 54O – 44C) and - 81.43º (43C – 48C – 53O – 49C) and showing a distance between 53O···54O of 2.679 Å. On the other hand, the distance between 36O···42O of the prenyl chain and the methoxy group attached to B-ring is 2.766 Å, which determines directly the orientation for methoxy groups. This correlates quite well with the results obtained by the X-Ray diffraction studies reported for similar structures [6].

The bond lengths for methoxy groups in chalcone **3** obtained with DFT:M06-2X/6-311++G(d,p) show average bond distances for O – C_aromatic _of 1.351 Å (54O – 43C = 1.339 Å, 53O – 48C = 1.355 Å and 42O – 37C = 1.358 Å), while for O – C_methyl _in methoxy groups the average bond distances is 1.407 Å (54O – 44C = 1.401 Å, 53O – 49C = 1.408 Å and 42O – 38C = 1.412 Å). These average values are lower than the reported data for chalcone derivatives, however, they show only a slight discrepancy with the values reported in the literature. The bond distances are: a) 1.375 Å for O – C_aromatic_, b) 1.421 Å for O – C_methyl _(both for *p-*methoxybenzyl 2α - methyl - 2β - [(R) - acetoxy(methoxy)methyl] - 6β - phenoxyacetamidopenam-3α-carboxylate [41]), and c) 1.373 Å for O – C_methyl _and d) 1.420 Å O – C_methyl _(for the compound 1-(2-hydroxy-4-methoxyphenyl)-3(2,3,4-trimethoxy-phenyl) prop-2-en-1-one [6]). According to the obtained results, the M06-2X functional in combination with a 6-311++G(d,p) basis set, reproduces experimental geometrical parameters with a good accuracy for bond distances and bond angles in chalcone derivatives.

### NMR analysis

#### ^1^H-NMR and ^13^C NMR experimental results

**Figure 3:**
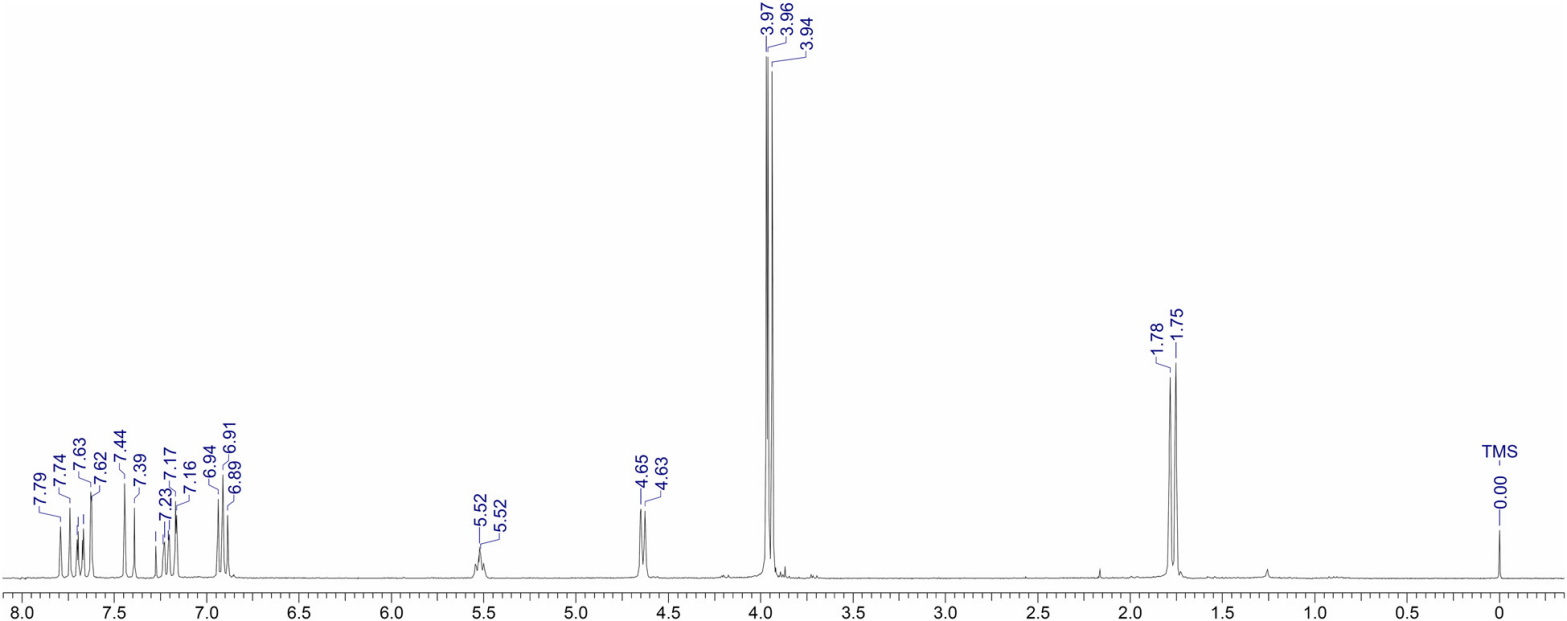
**Experimental **^**1**^**H-NMR spectrum for the new synthesized (2E)-1-(3,4-dimethoxyphenyl)-3-{3-methoxy-4-[(3-methylbut-2-en-1-yl)oxy]phenyl}prop-2-en-1-one (Compound 3).**

**Figure 4:**
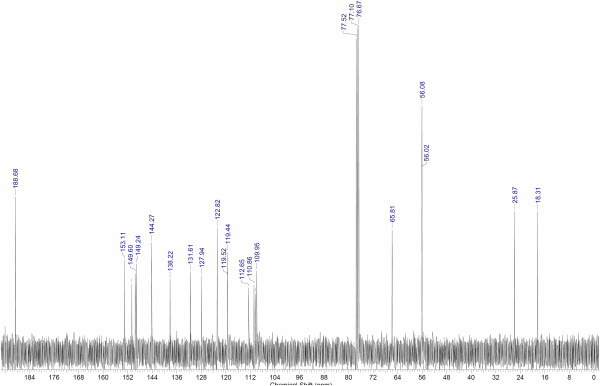
**Experimental **^**13**^**C-NMR spectrum for the new synthesized (2E)-1-(3,4-dimethoxyphenyl)-3-{3-methoxy-4-[(3-methylbut-2-en-1-yl)oxy]phenyl}prop-2-en-1-one (Compound 3).**

**Figure 5:**
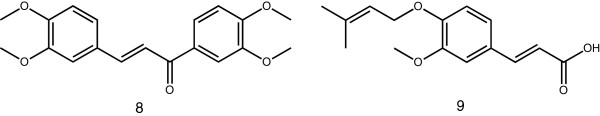
**Reference compounds used for **^**1**^**H and **^**13**^**C NMR assignment confirmation of chalcone 3.**

In Figures 3 and 4, the ^1^H-NMR and ^13^C-NMR experimental spectra for the new synthesized chalcone **3** are shown. The signals assigned for the chalcone were based both on the chemical shifts and coupling constants, for the proton, and on the chemical shift signals for the carbon spectra. These data were also compared with chalcone **8** (See Figure 5), which possesses the same substitution pattern on the aromatic rings [42-44].

The assigned and the compared data show an excellent agreement, thus confirming the structure for the obtained chalcone. The signals assigned for the prenyloxy side-chain were also correlated with the data obtained for boropinic acid **9** (Figure 5), a naturally-occurring prenyloxycinnamic acid, also showing an excellent agreement [45].

#### Comparison of theoretical methods for ^1^H and ^13^C experimental chemical shifts

Once assigned the experimental signals of the new compound, a comparison between the experimental and theoretical chemical shifts was carried out. In Tables 5 and 6 the values for ^1^H and ^13^C chemicals shifts are given. The theoretical values were obtained with the GIAO method at the Hartree-Fock and several DFT levels of theory, in combination with the 6-311++G(d,p) basis set (using the previously M06-2X/6-311++G(d,p) optimized geometry of compound **3** and TMS as reference).

**Table 5:** **Experimental and theoretical data for **^**1**^**H NMR chemical shifts in ppm, calculated for compound 3 in M06-2X/6-311++G(d,p) optimized geometry**

**Atom label**	**6-311++G(d,p)**						**Experimental data**
	**DFT:**	**DFT:**	**DFT:**	**DFT:**	**DFT:**	**HF**	
	**B3LYP**	**PBE1PBE**	**TPSS**	**M05-2X**	**M06-2X**		
**33, 34, 35**	1.66	1.62	1.73	1.69	1.66	1.61	1.76
**28, 29, 30**	1.73	1.71	1.82	1.42	1.51	1.66	1.79
**50,51, 52**	3.58	3.54	3.68	3.37	3.58	3.39	3.97
**45, 46, 47**	3.73	3.68	3.84	3.72	3.87	3.51	3.94
**39, 40, 41**	3.65	3.59	3.75	3.58	3.63	3.44	3.97
**23**	3.78	3.76	3.96	3.70	3.70	3.35	4.63
**24**	4.62	4.60	4.74	4.55	4.48	4.37	4.65
**26**	5.78	5.85	5.86	6.39	6.65	5.67	5.53
**18**	6.42	6.55	6.36	6.85	7.00	6.58	6.89
**4**	7.03	7.16	7.06	7.49	7.77	7.28	6.94
**6**	7.72	7.83	7.70	8.32	8.44	7.95	7.17
**5**	7.26	7.37	7.31	7.85	7.81	7.39	7.22
**11**	7.51	7.59	7.60	8.02	8.29	7.26	7.42
**17**	8.06	8.14	7.93	8.28	7.97	8.55	7.63
**16**	7.67	7.80	7.66	8.42	8.37	7.90	7.69
**9**	7.88	7.95	7.82	8.15	8.34	8.37	7.77
Unsigned difference average^*^	0.251	0.305	0.225	0.517	0.541	0.438	—

* Calculated as the difference between experimental and theoretical values.

**Table 6:** **Experimental and theoretical data for **^**13**^**C NMR chemical shifts in ppm, calculated for compound 3 in M06-2X/6-311++G(d,p) optimized geometry**

**Atom label**	**6-311++G(d,p)**						**Experimental data**
	**DFT:**	**DFT:**	**DFT:**	**DFT:**	**DFT:**	**HF**	
	**B3LYP**	**PBE1PBE**	**TPSS**	**M05-2X**	**M06-2X**		
**TMS**							
**C**	184.3	189.4	185.4	190.9	188.8	195.2	188.1^a^
**Chalcone 3**							
**19**	184.38	183.78	175.00	206.29	202.74	187.04	188.68
**43**	160.65	159.54	151.48	177.15	171.63	161.87	153.11
**21**	160.94	159.86	153.17	177.15	174.30	158.80	150.67
**37**	160.09	158.78	152.27	175.56	173.06	156.64	149.60
**48**	155.40	153.63	146.72	172.47	164.48	151.23	149.24
**8**	149.15	148.98	140.81	167.53	165.44	153.45	144.27
**31**	145.64	144.50	138.93	162.86	164.26	143.28	138.22
**12**	138.25	136.97	130.95	155.14	151.63	134.82	131.61
**1**	129.15	129.14	122.61	147.00	143.97	129.38	127.94
**13**	128.09	128.14	120.58	145.25	141.22	133.61	122.87
**10**	120.47	120.80	114.52	136.30	134.81	118.52	122.82
**7**	138.29	136.98	131.58	154.11	152.28	135.95	119.52
**25**	128.25	128.05	122.39	143.87	142.90	126.28	119.44
**2**	134.78	134.68	128.02	151.28	150.14	136.05	112.65
**3**	123.44	123.67	116.70	140.14	138.96	126.45	110.86
**14**	128.61	129.00	120.78	146.10	141.89	135.42	110.46
**15**	109.45	109.52	103.29	124.48	121.61	109.87	109.95
**22**	73.10	72.30	72.10	74.10	73.80	64.50	65.81
**49**	59.20	58.80	58.00	60.60	60.00	55.20	56.10
**44**	54.40	54.00	54.30	53.10	54.60	49.10	56.08
**38**	60.40	60.00	59.20	61.00	61.30	55.80	56.02
**27**	28.00	27.90	27.20	26.30	25.90	25.50	25.87
**32**	18.90	19.10	18.30	19.60	20.50	17.40	18.31
Unsigned difference average^*^	7.221	6.653	5.226	17.376	15.760	7.355	

^a^ Experimental value for TMS was taken from reference: Chem. Phys. Lett. **1987**, 134, 461–466.

* Calculated as the difference between experimental and theoretical values.

The shielding constants obtained with the GIAO method were found to converge almost to the same experimental value using a sufficiently large basis set. The convergence of the GIAO method with respect to the experimental data is adequate to predict chemical shifts, with a good accuracy for relatively large molecules such as **3**.

The comparisons shown in Tables 5 and 6 indicate that the DFT methods may predict good results when these are compared to experimental values, however, it can also be seen that these methods provide only a small improvement over the Hartree-Fock method, as can be observed in the ^13^C data where the chemical shifts are too deshielded with respect to the experimental values. The tendency in the GIAO calculations for ^1^H chemical shifts using DFT methods shows that the best method with an unsigned difference average of 0.225 ppm is the TPSS functional, followed in order of accuracy by B3LYP, PBE1PBE, HF, M05-2X and M06-2X, where all of them present an unsigned difference average below 0.541 ppm. The most popular functional B3LYP which is used frequently in NMR studies shows an unsigned difference average of 0.251 ppm. It can be noticed that in ^1^H NMR calculations the same behavior is exhibited for chemical shifts calculated with different methods (Table 5). Some of these obtained values are overestimated, especially, for hydrogen atoms located in B-ring–8C = 10C–19C–(20O)–A-ring segment.

On the other hand, in the ^13^C NMR calculations, (See Table 6) TPSS is the best method, with an unsigned difference average of 5.226 ppm followed by PBE1PBE, B3LYP, HF, M06-2X and M05-2X. The latter has the highest difference average value of 17.376 ppm. For other methods as PBE1PBE, B3LYP and HF, the obtained chemical shifts for ^13^C are roughly equal within a few ppm of difference; whereas the PBE1PBE and B3LYP functionals provide a small improvement over HF calculations. The calculated ^13^C NMR indicates that almost all the obtained values with HF and DFT-based methods were overestimated (when comparing with the experiment), and it seems to be no strong advantage of DFT over HF method. Nevertheless, the difference average shows that only two functionals give a difference higher than 10 ppm (M06-2X and M05-2X) for ^13^C NMR signals.

**Figure 6:**
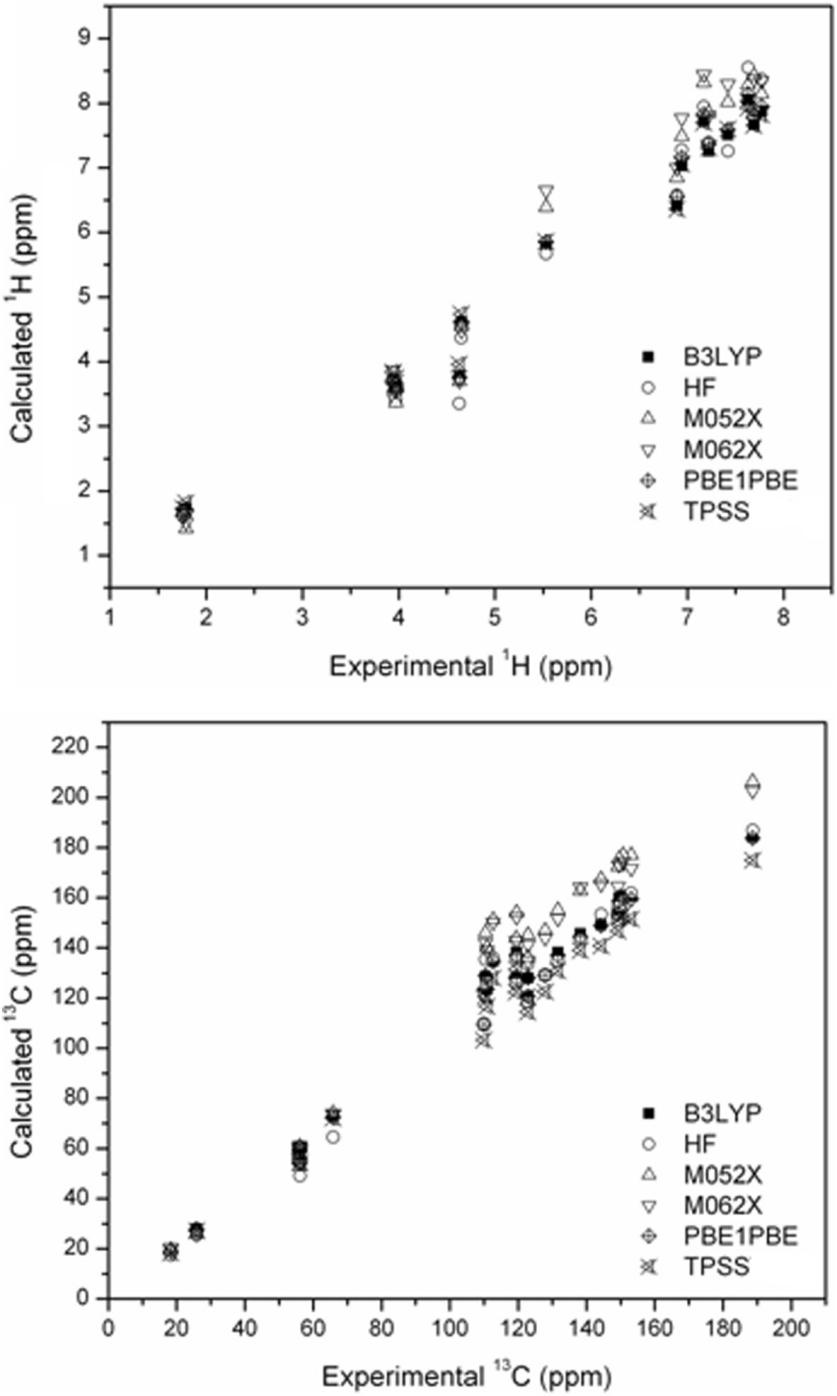
**Correlation plots for **^**1**^**H and **^**13**^**C NMR chemicals shifts of chalcone 3 calculated with theoretical methods vs experiment.**

In order to analyze with more detail the accuracy of the theoretical methods employed in the prediction of NMR chemical shifts for chalcone **3,** a Pearson's correlation statistical method was applied. Table 7 and Figure 6 show the obtained values for the correlation coefficient (*r*). As can be seen from these results, all the methods employed are in good agreement with the experimental values, however, the lowest values of the correlation coefficient were obtained for M05-2X and M06-2X DFT functionals. It can be noticed that the values of the correlation coefficient obtained with this methodology showed a good correlation for ^1^H NMR calculations (See Table 7). This could indicate that an accurate description of the molecular structure is necessary to achieve a more precise NMR theoretical data, since the chemical shifts are known to be quite dependent on the molecular geometry.

**Table 7:** **Correlation coefficient of the comparison between experimental and calculated **^**1**^**H and **^**13**^**C chemical shifts**

**Optimization//NMR**	***r***	
	^**1**^**H**	^**13**^**C**
M06-2X //B3LYP	0.990	0.989
M06-2X // PBE1PBE	0.990	0.989
M06-2X //TPSS	0.991	0.989
M06-2X //M05-2X	0.986	0.988
M06-2X//M06-2X	0.984	0.987
M06-2X //HF	0.982	0.985

Figure 6 illustrates the correlation between calculated and measured chemical shifts for ^1^H and ^13^C obtained with the GIAO formalism in the M06-2X/6-311++G(d,p) optimized geometry. It is important to note that the functionals with the best performance for geometry optimizations (M05-2X and M06-2X) do not present good results in the prediction of NMR values, especially for ^13^C NMR signals. However, all the tested methods showed high values for the correlation coefficient, with an excellent performance-to cost ratio in molecules with similar structures.

## Conclusions

In this work, a novel chalcone derivative was synthesized: (2E)-1-(3,4-dimethoxyphenyl)-3-{3-methoxy-4-[(3-methylbut-2-en-1-yl)oxy]phenyl}prop-2-en-1-one (chalcone **3**); the compound was also characterized by NMR. For a detailed molecular structure description some quantum-chemical calculations were performed with several HF and DFT methods. The comparison of the theoretical results with experimental X-ray diffraction data for similar compounds, showed that, from all the tested methods, the best performance for geometrical parameters in chalcone derivatives was for M06-2X (in combination with a large basis set 6-311++G(d,p)). The geometrical study indicates that the presence of different substituents have little effect on the geometric parameters in the main structure of chalcones. Additionally, these parameters are well reproduced by theoretical calculations for structurally similar compounds.

According to our results, the methodology employed for NMR calculations show a good performance in the accuracy of the predicted signals, mostly when NMR calculations were made using a optimized geometry obtained with M06-2X/6-311++G(d,p). This fact indicates that a good description of the molecular structure is necessary to obtain more accurate values. It was found that the calculated ^1^H and ^13^C chemical shifts correlate quite well with the experimental data for all tested methods. However, the best outcome for theoretical NMR values was for the DFT:TPSS method in the geometry optimized at M06-2X/6-311++G(d,p). The Pearson's correlation coefficient showed that all the tested methods are in good agreement with the experimental data, while the unsigned difference average between experimental and theoretical NMR values follows the order TPSS ≈ B3LYP < PBE1PBE < HF < M05-2X ≈ M06 -2X for ^1^H chemical shifts, and for ^13^C chemical shifts the order is TPSS < PBE1PBE < B3LYP ≈ HF < M06-2X < M05 -2X.

The theoretical information collected in this study can be further used in the analysis of the molecular geometry and in the more detailed assignments of experimental NMR signals for either natural or synthetic molecules containing similar structures.

## Competing interests

The authors declare that they have no competing interests.

## Authors’ contributions

JCEH and AACD carried out the synthesis and NMR characterization of synthesized compound, NRFH, DGM and LMRV implemented and carried out the computational studies. AACD, GVNM and LMRV drafted the manuscript. All authors read and approved the final manuscript**.**
